# The transition from in-person to virtual museum programing for individuals living with chronic pain – A formative evaluation

**DOI:** 10.1017/cts.2022.392

**Published:** 2022-04-19

**Authors:** Ian J. Koebner, Helen J. Chatterjee, Claudia M. Witt, Daniel J. Tancredi, Ruchi Rawal, Gary Weinberg, Frederick J. Meyers

**Affiliations:** 1 Department of Anesthesiology and Pain Medicine, University of California, Davis, USA; 2 Department Genetics, Evolution and Environment, UCL Biosciences, University College London, UK; 3 Institute for Complementary and Integrative Medicine, University Hospital Zurich and University of Zurich, Zurich, Switzerland; 4 Department of Pediatrics, Center for Healthcare Policy and Research, University of California, Davis, USA; 5 Center for Healthcare Policy and Research, University of California, Davis, USA; 6 Division of Hematology and Oncology, University of California, Davis, USA

**Keywords:** Museum, virtual museum, research, chronic pain, social connection

## Abstract

Museum engagement may be an effective approach for decreasing social disconnection and pain among individuals living with chronic pain. In October 2019, we launched a randomized controlled trial to assess the feasibility of museum engagement for individuals living with chronic pain; the study was halted in March, 2020 due to Covid-19-related safety concerns. This paper describes the process of transitioning from in-person to virtual museum programing in order to continue the study. Virtual museum programing is a feasible option for individuals living with chronic pain that is amenable to research and which may improve accessibility, inclusivity, and scalability relative to in-person programing.

## Introduction

Pain is a complex biopsychosocial phenomenon [1–4], defined by the International Association for the Study of Pain (IASP) as “an unpleasant sensory and emotional experience associated with, or resembling that associated with, actual or potential tissue damage” [5]. Population-based estimates of chronic pain among US adults range from 11% to 40% [6] and approximately 8% of US adults (roughly 20 million individuals) live with high-impact chronic pain that frequently limits life or work activities [7].

Chronic pain puts individuals at increased risk of social disconnection and reduced social role functioning, both of which negatively impact pain interference and pain intensity over time [8]. From a patient perspective, disrupted social roles and relationships are among the most distressing aspects of living with chronic pain [9] and participation in social and family activities is among the most valued outcomes of chronic pain management [10]. While group-based therapy and supportive programs for individuals living with chronic pain, such as cognitive behavioral therapy and mindfulness-based stress reduction, may be potentially helpful interventions for chronic pain management [11], and can be considered “social” in so far as they involve relating to and interacting with other people, these programs do not center sociality, often take place in a clinical context which can be stigmatizing [12], and rarely target or evaluate social outcomes [13]. The Covid-19 pandemic has only exacerbated the vulnerability of individuals living with chronic pain to social disconnection and underscores the need for creative solutions [14].

While a number of non-clinical community activities could reduce social disconnection [15,16], museums may be a uniquely valuable public health partner in addressing chronic pain. There are 35,000 museums in the USA [17] and many have websites that provide continuous virtual access to their collections, making these digital collections accessible to many who would not otherwise be able to visit in person. In addition, there is no admission cost at the majority of US federal museums, and many other museums have reduced fees for vulnerable populations [18]. Virtual museum engagement, which is free, available 24 h a day, unrestricted by geography, and more accessible to those with physical disabilities than in-person visits, may create a favorable context for public health programing [18]. The addition of interactive virtual museum programing may add value to these static options by creating innovative social environments where individuals can sustain or initiate connections to both other participants as well as to the museum and its collection. Furthermore, because museums neither diagnose nor treat health problems they may be experienced as less stigmatizing [18,19]; an attribute that may be particularly beneficial to individuals living with chronic pain who often experience internalized stigma [20] or face stigmatization from their health care providers [12,21]. A significant evidence base exists for the role the arts can play in promoting health and managing illness across the lifespan [22]. In particular, arts organizations may be valuable public health partners in both preventing the development of chronic pain [23] and in addressing the social disconnection that accompanies chronic pain [18,24].

However, further research is needed in this area. The National Endowment for the Arts (NEA) published a literature review on the arts, pain management, and substance use disorder in 2020 [24] that identified 79 studies on the topic of pain management. Seventy-two (91%) studies evaluated a music-based intervention and 46 (58%) targeted individuals with postoperative pain. A key finding of the review is the need for research conducted in non-hospital settings that target the social aspects of living with chronic pain [24]. Considering the potential that museums may facilitate a sense of social connection and that social connection may be analgesic [25–28], we hypothesized that specialized museum engagement offered to individuals living with chronic pain may decrease perceived social disconnection and pain.

The Integrative Pain Management Program at the University of California, Davis (UCD) and the Crocker Art Museum, located in Sacramento, CA, formed a partnership in 2014 to establish the first museum-based program for individuals living with chronic pain [19,29]. Both healthcare and museum professionals involved in the program reported that it added value to their respective missions [29] and program participants expressed satisfaction with various dimensions of the program, from registration to the quality of the overall experience [19]. Details on this partnership development have been previously published [29].

To assess the feasibility of museum engagement as a treatment approach for individuals living with chronic pain, we designed a randomized controlled trial to describe the joint and separate effects of two types of museum experiences, Art Rx (museum experience 1) and Artful Meditation (museum experience 2). The study required that individuals met the following inclusion criteria – ≥18 years of age; English speaking; live with chronic pain (≥6 months); have moderate pain or greater (≥4/10 on a Numerical Rating Scale, range of 0 [no pain]–10 [worst pain imaginable], in response to the question "Over the past week what was your average pain intensity?"); be moderately lonely or greater (Score of ≥4 on the 3-item Loneliness scale [30], range of 3–9), and have access to the internet and a web-browsing device. The study’s exclusion criteria were previous participation in an Art Rx tour or Artful Meditation program; living with Dementia or Alzheimer’s disease. Full details of the RCT’s study design are available on its ClincalTrials.gov registration page – (NCT04091893).

Art Rx consists of docent-facilitated group discussion, either in-person or virtually, about selected objects of art. Artful meditation combines guided mindfulness meditation instruction with a series of art appreciation exercises in the context of an art gallery or virtual art space. Both programs aim to build a sense of social connection among participants and to create meaningful cognitive and emotional experiences. Both Art Rx and Artful Meditation can be conceptualized as complex interventions [31], in that they have multiple and interacting components, require a number of behaviors by those delivering and receiving the program, target a variety of groups, and allow for flexibility and tailoring of the program. The ability to develop, adapt, and evaluate complex evaluations requires a thorough understanding of how these program are implemented [31]. Therefore, to improve the completeness of reporting, and ultimately the replicability of Art Rx and Artful Meditation, the authors created user guides and fidelity checklists for both programs (see Supplementary Material) using an adapted version of the 12-item Template for Intervention Description and Replication (TIDieR) [32].

The UCD Institutional Review Board (IRB) approved this study (IRB # 1415639-4). Recruitment began on October 12, 2019 with a target sample of 64 individuals. Due to Covid-19-related safety concerns, we stopped enrollment and suspended the trial in March 2020 with 24 individuals enrolled. The following section describes the process undertaken to transition to and deliver the virtual museum programing.

### Transition Process

On March 11 2020, the World Health Organization declared the Covid-19 viral disease a pandemic [33], present in at least 114 countries and responsible for the deaths of more than 4,000 people. The next day the UCD IRB administration recommended that study investigators take specific actions to limit transmission of the virus by delaying or otherwise modifying non-essential interactions. In particular, the IRB administration recommended that research involving group meetings or appointments consider delaying or using alternative interactions via electronic means if possible. In-person museum programing was immediately suspended.

On March 17, the UCD research team met to discuss the options for managing the museum study: (1) temporarily suspend the trial with the hope of resuming in-person programing; (2) end the study and use data collected to date; (3) transition to virtual museum programing and resume trial. The research team chose to transition to virtual museum programing, given its potential to create and preserve meaningful social connections despite physical distancing measures. From March 17 through May 9, 2020, the UCD research and Crocker Art Museum teams met at least weekly to refine the process for virtual museum programing through an informal consensus model of decision-making among organizational stakeholders. (See Table 1. Timeline and summary of transition to virtual museum programing)

**Table 1.** Timeline and summary of transition to virtual museum programing

March 12th, 2020	University of California, Davis (UCD) and Crocker Art Museum suspend in-person programing and study.
March 17th, 2020	UCD Research Team meets to discuss options: Temporarily suspend study End study and use data collected to date. Transition to virtual museum programing and resume trial Next steps: Discuss feasibility of transition with Crocker Art Museum Consult with advisors in museum programing and research design about best practices
March 17–April 7, 2020	UCD team clarifies preference to transition to virtual programing and resume trial as soon as possible. The UCD team meets three times to discuss logistics of virtual programing. Study inclusion/exclusion criteria are refined to assure participants have access to the internet and a web-browsing device.
April 7th, 2020	UCD Team meets with partners from the Crocker Art Museum to discuss the museum’s interest and capacity for virtual programing. UCD and Crocker Art Museum decide to move forward with virtual programing. Roles: Crocker Art Museum – Program facilitation and content development UCD – Technical facilitation and data collection (e.g. setting up hyperlinks for program participation, creating the online meeting site, administering surveys and collecting data from research participants)
April 14, 2020	Weekly UCD/Crocker Art Museum meetings to refine the virtual programing process (e.g., registration, program and technical facilitation, and data collection) Collaboratively create a “virtual culture guide” to help assure an engaged and prosocial program experience. Update fidelity checklist to the virtual program environment to assess integrity of program implementation.
April 23, 2020	Practice virtual program #1 Attendees include research and museum team members (i.e. no research participants)
April 28, 2020	Debrief practice virtual program. Unanimous agreement that virtual program is functional and that the team is ready to resume research study.
May 9, 2020	First small-scale virtual program for select research participants. N = 7
June 13, 2020	First virtual program open to the public N = 22
July–September, 2020	Launch third party social media campaign to recruit participants

Key outputs from these meetings include:

1. **Choice of platform:** The research team chose to use a cloud-based video service as the platform for the virtual museum programing. The team considered a static video of the museum gallery with an accompanying docent talk, but felt this option was not an appropriate alternative for in-person programing since it creates a passive and individual participant experience that does not allow for social interaction. Multiple cloud-based video services exist. To select a cloud-based video service, the research team considered the following items:
  - **Security and Privacy.** Does the platform provide encryption, administrator control over participant access, security of any stored recordings, the option for password protected meetings, and, if relevant, compliance with the Health Insurance Portability and Accountability Act (HIPAA) standards for the protection of sensitive patient data?
  - **Accessibility, Usability, and Inclusion.** Does the platform comply with national and international accessibility standards (e.g. Americans with Disabilities Act and Web Content Accessibility Guidelines)? Is the platform accessible and usable given participants’ varying technical abilities? Can the software be accessed from a variety of electronic devices (e.g. tablet, smartphone, computer)? Can users join meetings with a single click and with no external software requirements? The study team chose a platform that was accessible by computer, tablet, and smartphone, and was compliant with national and international accessibility standards. The platform had, among other features, closed captioning, post-meeting transcripts, and the capability for participants to control their experience with just the keyboard.
  - **Quality of the audio/video.** Can participants both see and hear one another clearly? What is the image quality of the video and is it sufficient to showcase works of art?
  - **Number of participants.** How many participants can participate in any given meeting?
  - **Additional features.** Does the platform allow for screen and/or document sharing, breakout rooms, instant chat messaging, artificial intelligence transcription, third-party application integration, etc.?
  - **Cost.** What is the fee structure for the platform – number of hosts, number of participants, duration of meetings, additional video conferencing features (e.g. transcription, breakout rooms, etc.)?
  - **Customer support:** Does the platform offer easy and comprehensive customer support?
2. **Delegation of roles.** The Crocker Art Museum team assumed responsibility for program content (e.g. selection of artwork, program facilitation). The UCD team assumed responsibility for logistics (e.g. creating and disseminating hyperlinks for participation, assisting participants with technical issues) and research (e.g. survey administration and data collection).
3. **Optimization of the user experience.** The UCD and Crocker Art Museum teams developed a ‘virtual culture guide’ to help assure meaningful and prosocial interactions during program implementation. This guide included the following suggestions for the program facilitators to share with participants:
  - Recognize the difficult and unprecedented nature of these times and that virtual gatherings, such as this one, represent a new way of interacting for many.
  - State the goal of the program: to create a positive social experience for all participants. To achieve this goal, make several suggestions with demonstrations as appropriate:
    - Mute microphone when not speaking
    - Show how to change ‘views’ (e.g. gallery view, speaker view, etc.)
    - Avoid cross talk; one person talks at a time
    - Use the chat function if technical or other questions arise. The chat function allows the facilitators to respond to questions without program interruption.
    - Invite and encourage participants to turn their video camera on so that they can be seen. Underscore that a primary intention of this program is to create community and group participation just like in an actual museum. Therefore, being able to both hear and see participants is valuable. However, comfort is paramount; if for any reason participants prefer not to use video support their decision to turn it off.
    - Encourage participants to move or change position during the experience to make themselves comfortable.
4. **Data collection process.** UCD team sent participants links to pre- and post-surveys immediately before and after their virtual museum experience, respectively. Surveys were completed online and research assistants were available via email or phone to help participants complete their surveys as needed. To assess the individual and comparative feasibility and acceptability of the in-person and virtual museum programing, the research team also collected socioeconomic and demographic data as well as program satisfaction survey data from all participants. In addition, semi-standardized interviews were conducted with both in-person and virtual museum program participants to explore the perceived impact of the in-person and virtual museum experiences on the lived experience of pain as well as the perceived strengths and opportunities of the programs. Finally, a research assistant completed a fidelity checklist during every museum experience, both in-person and virtual, to assure that key components of the program were adhered to as well as to give the research assistant an opportunity to reflect openly on participant engagement. (Analysis of these surveys and interviews is underway and will be reported at a later date.)
5. **Beta testing of the virtual museum programing.** A small number of UCD researchers and Crocker Art Museum personnel were invited to attend and provide feedback on several practice virtual museum experiences.

Once the research and museum team members reached consensus that the programing and data collection logistics had been optimized the trial resumed on May 9, 2020. Virtual museum programing was held on the second and fourth Saturday of every month. To facilitate recruitment, the research team hired a third party to develop a social media campaign for the study (July–September, 2020).

## Discussion

Socially based interventions targeting individuals living with chronic pain are limited [3,13], and both the cultural sector broadly [24,34], and museums in particular [19,29] may have a valuable role to play in addressing the social disconnection that is associated with chronic pain. This paper describes the transition from an in-person to virtual museum study in response to Covid-19 social-distancing safety measures. From a research perspective, we found virtual museum programing amenable to data collection procedures (e.g. online survey administration) and research staff appreciated the increased automation it afforded (e.g. scheduled emails sent to participants with links to online surveys versus administering hard-copy surveys at the museum gallery to each research participant). The institutional and implementation burdens associated with the transition from in-person to virtual museum programing described in this paper were manageable. The transition required approximately eight meetings centered on logistics and four beta-tests of the virtual museum programing with a total of 58 days elapsing between the conclusion of the in-person museum study and the start of the virtual museum study.

Virtual museum programing may also be a mechanism to help museums realize their commitment to diversity, equity, inclusivity, and accessibility as it affords museums a reach and scale that is not possible with in-person programing. For example, participation in virtual museum programing is not bound by an individual’s geography and is accessible to the homebound and hospitalized. However, virtual programs also have the potential to exacerbate health disparities because socioeconomically and medically advantaged individuals may be more likely to participate in and therefore benefit from them [35]. A limitation of this study was its inability to thoroughly address digital and health equity concerns associated with a transition from in-person to virtual programing due to the unplanned, unfunded, and time-sensitive nature of the transition. For example, we did not have resources to provide the training or technology (e.g. computer and internet connection), either directly or through community partnerships, required to participate in the virtual museum programing. Therefore, part of this study’s eligibility criteria was access to the internet and a web-browsing device. Furthermore, while individuals with the lived experience of pain were included in the development of the original partnership and in-person programing [19,29], they were not included in the transition process. While a comprehensive review of digital and health inequities along with strategies to mitigate them are beyond the scope of this paper, future virtual museum programs studies should acknowledge and integrate the extended timeframe, resources, and community partnerships required to create accessible and inclusive programs that serve a representative population [36].

Virtual museum programing has additional limitations. First, online interactive programing is susceptible to malware and hacking attempts. Authentication measures like CAPTCHA (Completely Automated Public Turing test to tell Computers and Humans Apart) can be used to help assure that users are humans. For this study, a research assistant made personal contact with all research participants. This contact not only served to confirm the authenticity of participants but also allowed the research team to clarify the research process and answer any outstanding questions. Second, because participants in virtual programing can leave with a simple key stroke on their electronic devise, there is a greater theoretical risk of attrition then in-person programing which inherently requires greater effort on the part of participants to both attend and leave. Future mixed methods research is needed to explore the effectiveness of virtual museum engagement and the comparative effectiveness of in-person versus virtual museum engagement. While virtual museum programing may be a welcomed alternative to in-person programing in certain contexts (e.g. among populations with restricted physical ability or access to a museum), we imagine that in many cases virtual programing will be a complement to in-person experiences allowing museums to expand their reach and impact.

The closure of museums around the world due to Covid-19-related safety measures was a loss for museum visitors and professionals alike. This paper describes how these social distancing measures created the impetus for our research team and museum partner to successfully transition to virtual museum programing. This transition allowed the public to continue to engage with the museum throughout the Covid-19 pandemic and for our research team to resume our RCT assessing the feasibility of both in-person and virtual museum programing to address social disconnection among individuals living with chronic pain. The process detailed in this paper for transitioning to virtual museum programing may be generalizable to programs that target other nontraditional museum visitors and therefore serve as a model for museums and other arts and cultural organizations wishing to expand their reach into the virtual space.

## Supplementary material

For supplementary material accompanying this paper visit https://doi.org/10.1017/cts.2022.392. click here to view supplementary material
