# Supplementary material for: The transition from in-person to virtual museum programing for individuals living with chronic pain – A formative evaluation
Source: J Clin Transl Sci. 2022 Apr 19;6(1):e58. doi: 10.1017/cts.2022.392 (PMC9161051; doi:10.1017/cts.2022.392)
Supplement: Supplementary file 1 [file S2059866122003922sup001.docx]

**ART RX & ARTFUL MEDITATION:**

**USER GUIDES AND FIDELITY CHECKLISTS**

**Introduction**

The idea that the creative arts can contribute to health and wellbeing is not new.^1^ For thousands of years, in widely divergent global cultures, this idea has been expressed and manifested in a myriad of ways. But despite a growing literature supporting the positive contribution the arts can play in health and well-being the value of art in health in the 21^st^ century is widely underappreciated and under-researched.^2^

Of the many conditions that could benefit from art-based interventions, chronic pain may be one of the most suitable. Chronic pain is a leading cause of morbidity and disability across the world,^3^ affecting tens of millions of adults in the United States alone.^4^ More importantly, pain is a complex biopsychosocial phenomenon,^5-9^ that is always a personal experience influenced to varying degrees by biological, psychological, and social factors.^10^ For example, alleviating social disconnection has been demonstrated to reduce pain,^11-13^ yet interventions targeting social disconnection in individuals with chronic pain are uncommon.^5-8,14^

Arts-spaces can be valuable public health partners in addressing the social disconnection that accompanies chronic pain^15^ and there is evidence that cultural engagement, including museum attendance, is protective against the development of chronic pain.^16^

Art Rx and Artful meditation were created to support the lived experience of chronic pain with a set of structured activities amenable to research and that can be conducted either in-person or virtually. These activities fall in the realm of “art-in-health” rather than “art therapy.” Art therapy has been validated as a supportive treatment for a variety of symptoms, age groups, and disorders.^17^ But whereas art therapy is typically conducted individually or with small groups led by a trained art therapist, Art Rx and Artful Meditation offer a broader use of art, for both small and large groups, and involve a range of leaders/facilitators that are not healthcare professionals (although the incorporation of art therapists or art therapy techniques could be perfectly appropriate and/or helpful).

Arts-spaces (e.g., museums, galleries, art-related organizations, community centers) possess several characteristics that may make them well-suited public health partners^15^: as non-clinical organizations that neither treat nor diagnose they may be less stigmatizing than health care organizations and arts-spaces often offer free or reduced admission fees for special populations, thereby decreasing obstacles to access and increasing the likelihood of regular attendance.

Arts in health programs like Art Rx and Artful Meditation can be conceptualized as complex interventions,^18^ in that they

- Have multiple and interacting components
- Require a number of behaviours by those delivering and receiving the program
- Target a variety of groups
- Have variability in outcomes
- Allow for flexibility and tailoring of the program

The ability to develop, adapt and evaluate complex evaluations requires a thorough understanding of how these program are implemented.^18^

**The TIDieR Checklist**

To improve the completeness of reporting, and ultimately the replicability, of interventions, an international group of experts and stakeholders in 2014 developed the 12-item Template for Intervention Description and Replication (TIDieR) checklist and guide.^19^ The emphasis of the checklist is on clinical trials, but the guidance is intended to apply across all evaluative study designs with a goal of improving the reporting and evaluation of interventions. Intervention descriptions, especially for complex interventions such as Art Rx and Artful Meditation, involve more than providing a label or an ingredients list. Key features—including duration, dose or intensity, mode of delivery, essential processes, and monitoring—can all influence efficacy and replicability but are often missing or poorly described. The TIDierR format has been chosen as the framework for this project both to facilitate any research activities that might be conducted using the Art Rx and Artful Meditation modules and, also, to ensure a high level of transparency, completeness, and consistency in the use and evaluation of the modules by museums, arts organizations, community groups, and any other individuals interested in using art to support individuals living with chronic pain.

The TIDieR format and the Art Rx and Artful Meditation guides below use the following structure:

**Brief Name**

A name or phrase describing the intervention

**Why?**

The rationale, theory, or goals underpinning the intervention or the components of a complex intervention.

**What?**

A full description of the physical and informational materials used in the intervention, and the procedures, activities, or processes used.

**Who Provided?**

A description of the leaders, facilitators, or providers who organize and lead an intervention and any expertise, training or qualifications required.

**How?**

A description of the modes of delivery (e.g., in-person, virtual, telephone), whether the intervention is provided to individuals or groups, size of groups, and any other delivery features essential to the working of the intervention.

**Where?**

Description of the location(s) in which the intervention is to be conducted and any features or infrastructure about the location relevant to the delivery of the intervention.

**When and How Much?**

The number of times an intervention is delivered, over what period of time, including number of sessions, their schedule, duration, and intensity.

**Tailoring**

Description of any personalization, titration, or adaptations of an intervention. (Note: because neither Art Rx nor Artful Meditation involve this kind of personalization, this section is not included in any of the module-specific guides.)

**Modifications**

Description of any modifications of content or delivery that is either planned, or unplanned.

**How Well?**

How the fidelity of and intervention is to be evaluated, i.e., the degree to which an intervention happened in the way the organizers intended it to. Module-specific fidelity checklists are provided to help ensure uniform and consistent program evaluation.

**Who is this guide for?**

The Art Rx and Artful Meditation guides provide a framework for individuals wishing to create meaningful, potentially therapeutic experiences to support individuals living with chronic pain.

**Using the guide**

Each module comes with user guide and fidelity checklist. The user guide is written for the module facilitator(s). It offers an easy-to-read step-by-step guide for putting the module into practice. It is essentially a “how to” manual for each module. The fidelity checklist helps to assure the integrity of the program by allowing an observer (not the facilitator) to check if key features of the module were adhered to or if there were deviations. Fidelity is important for quality improvement and for research.

**ART RX – USER GUIDE**

**Brief Name**

Art Rx: Museum Tours For Individuals With Chronic Pain

**Why?**

Chronic pain is a complex biopsychosocial phenomenon. Individuals with chronic pain often experience loneliness, social disconnection, and stigmatization. Arts engagement can facilitate a sense of social connection as well as meaningful cognitive, emotional, and social experiences that may reduce the burden of chronic pain.

The goal of Art Rx is to reduce the burden of chronic pain by addressing the social disconnection and loneliness that often accompanies the condition.

There are four main steps to the Art Rx tour: (1) Welcome and introduction; (2) silent looking at the artwork; (3) docent-facilitated discussion; (4) a brief conclusion. These components are expanded in the next section. Tours last about 1 hour and 15 minutes and typically include 2 objects of art.

**What?**

Materials

Art Rx requires that all participants have a comfortable place to sit. At the Crocker Art Museum in Sacramento, California, which is where the Art Rx program was first developed and evaluated, every participant was provided with a lightweight stool. They are encouraged by the docents to adjust their position (e.g. sit and/or stand) to be comfortable whether the program is offered in-person or virtually.

Procedures

The four steps to the Art Rx tour are detailed below:

1. Welcome and introduction.

Inform participants that:

- 1. the museum is a place for all community members and that everyone is always welcome and encouraged to enjoy this community resource.
  2. the simple goal for today is to enjoy art, the museum space, and each other’s company.
  3. no art experience is needed, there are no right or wrong answers, everyone’s ideas and viewpoints are equally valid
  4. the program has been designed to be as comfortable as possible but if you need anything please feel free to approach one of the docents
  5. the tour will be about 1 hour and 15 minutes and that they are free to enjoy the museum for the rest of the day

1. Silent looking at artwork #1:
   1. Take 1 or 2 minutes to simply look at the artwork in silence; encourage participants, as needed, to come up for a closer look. In virtual programs, the facilitator shares an image off an artwork on their screen.
2. Docent-facilitated discussion:
   1. Used open-ended questions
   2. Paraphrase responses appropriately

Example: Docent: “What do you see?”

Participant: “I see a woman seated at a table.”

Docent repeats or paraphrases the participant: “Susan sees a woman at a table.” And then states, “What else is going on?” or “What else do you see?”

- 1. Break group into dyads/triads – In-person programming only
     1. Docent informs participants that they will now break into groups of 2 or 3 to have a breakout discussion and then we will come back to the group space.
     2. Docent asks everyone to "turn to the person on your right" to form a dyad, and adds, "groups of three are fine, too."
     3. Docent instructs dyads/triads to take turns telling the other person (people) their response or reaction to a specific question or prompt. After the first person shares, then the other person/people reciprocate. Sample prompts include:
        1. “How has your experience of or reaction to this piece of art changed between when you first saw it and now?”
        2. “Can you think of something in your life that this piece of art makes you think of - an experience, an object, maybe even a feeling it brings up for you?”
     4. Docent then asks people to come back together as a group, and ask if anyone would like to share some of what they had talked about.
  2. Conclude discussion, object #1:
     1. Docent either offers some information about the object of art or the artist, or generally summarizes all the different ideas that the participants have brought up.
  3. Docent repeats steps 2 and 3 for the next art object and as many other pieces of art as may be appropriate for the group.

1. Brief conclusion:
   1. Docent thanks everyone for coming.
   2. Reminds participants of the date of the next program (if any) and encourages them to attend.

**Who Provided?**

Art Rx tours at the Crocker Art Museum have been facilitated by museum docents or museum staff who received additional training in the Art Rx program:

1. Observation of a minimum of two (three is preferred) Art Rx tours
2. Lead one (maximum of two) mock Art Rx tours to be evaluated by the program administrator and a minimum of two other Art Rx leaders
   - 1. Mock tours will be composed of approximately five docents standing in as Art Rx guests.
     2. Meet with evaluators after the mock tour for feedback and acceptance, rejection or contingent approval into the Art Rx program as a docent.
     3. Those who receive contingent approval may be required to lead another mock tour or be able to address specific criticisms before acceptance into the program as an Art Rx docent.

Generally, Art Rx facilitators should be familiar leading discussions about objects of art with diverse groups in terms of physical ability and educational background. Facilitators leading virtual programs must be practiced in the use of the selected online forum and be able to, for example, assist participants with getting online and adjusting their audio and video settings.

**How?**

Art Rx tours are either in-person group experiences in a museum’s gallery space, or virtual online programs accessed via video-conferencing web-based platform. Group size varies. We recommend no less than 4 participants and no more than 20 participants in a group.

**Where?**

Art Rx tours can take place at any type of art museum, gallery, or non-traditional art-space with objects of art, or tours can be virtual, hosted on a web-based video-conferencing platform. For virtual tours, the overall format and structure are identical to in-person programs, although the dyad/triad discussions may be omitted. Rehearsals to ensure the integrity of the technology and facilitator comfort with the chosen platform are highly recommended for virtual programs.

**When and How Much?**

Art Rx tours can be scheduled as frequently or as rarely as staff and resources permit. Consider the needs of your participants. Scheduling programs on both weekdays and weekends can accommodate the varying routines and obligations of participants. Unless the demand for Art Rx programs becomes extremely high, there is typically no need to limit how many programs an individual can attend.

**Modifications**

Art Rx docents select different art works for each Art Rx tour. Allowing Art Rx docents to choose the art works viewed facilitates their sense of agency and enthusiasm for the program. Selecting different objects of art for each tour helps to assure the diversity and inclusivity of the program by appealing to the widest possible of range of art. Tour themes should be publicized well in advance if possible (i.e., at least a month) prior to any particular tour.

**How Well?**

It is recommended, but not required, that every Art Rx program be evaluated in a structured way, using a “Fidelity Checklist.” This can provide invaluable data on which to base refinements to the program, its content, and its presentation. An assistant to the facilitating docent could use the Art Rx Fidelity Checklist to record adherence to recommended steps and processes.

**ART RX FIDELITY CHECKLIST**

**Completed by:**

**Date:**

1. **Welcome and Program Introduction**

- Introduced research partnership with UC Davis
- This experience is for everyone: inform participants that this experience is for all community members and that everyone is always welcome and encouraged to enjoy this community resource
- The simple goal for today is to enjoy art and each others’ company
- No art experience is needed
- There are no right or wrong answers
- Everyone’s ideas and viewpoints are equally valid
- We want you to be as comfortable as possible so please feel free to get up and move and if you have a question or need anything please feel free to ask or send a chat message to one of the facilitators
- The experience will be about 1 hour and 15 minutes

*The Art Rx Docent introduced participants to the program’s goal?*

YES / NO

*The Art Rx Docent was welcoming?*

YES / NO

1. **Silent Looking at Art Work #1**

- Take 1 or 2 minutes to simply look at the artwork in silence.

*The Art Rx Docent gave participants an opportunity to silently look at the first object of art*

YES / NO

1. **Docent-facilitated discussion, Art Work #1**

- Docent used open-ended questions to encourage dialogue.
- Docent paraphrased responses appropriately.
- Docent encouraged everyone to speak
- Docent was positive and accepting of all voiced perspectives on the art

*The Art Rx Docent facilitated an inclusive discussion among participants about the first object of art*

YES / NO

1. **Conclude discussion on Art Work #1:**

- Docent either offered some information about the object of art or the artist, or generally summarized the different ideas that the participants brought up during discussion.

*The Art Rx Docent concluded discussion about object 1 by offering some information about the art work/artist and/or summarizing the comments made during the discussion*

YES / NO

1. **Silent Looking at Art Work #2**

- Take 1 or 2 minutes to simply look at the artwork in silence.

*The Art Rx Docent gave participants an opportunity to silently look at the first object of art*

YES / NO

1. **Docent-facilitated discussion, Art Work #2**

- Docent used open-ended questions to encourage dialogue.
- Docent paraphrased responses appropriately
- Docent encouraged everyone to speak
- Docent was positive and accepting of all voiced perspectives on the art

*The Art Rx Docent facilitated an inclusive discussion among participants about the first object of art*

YES / NO

1. **Conclude discussion on Art Work #2:**

- Docent either offered some information about the object of art or the artist, or generally summarized the different ideas that the participants brought up during discussion.

*The Art Rx Docent concluded discussion about object 2 by offering some information about the art work/artist and/or summarizing the comments made during the discussion*

YES / NO

1. **Program Conclusion**

- Docent thanked everyone for coming
- Docent reminded participants of the date of the next program and encourages them to attend

The Art Rx Docent(s) concluded the program and encouraged participants to enjoy the museum

YES / NO

**ARTFUL MEDITATION – USER GUIDE**

**Brief Name**

Artful Meditation: Meditation and Art Appreciation For Individuals Living With Chronic Pain

**Why?**

Chronic pain is a complex biopsychosocial phenomenon. Individuals with chronic pain often experience loneliness, social disconnection and stigmatization. Museums and arts engagement can facilitate a sense of social connection as well as meaningful cognitive, emotional and social experiences that may reduce the burden of chronic pain. In addition, even brief training in mindful meditation has been shown to reduce the burden of pain.

The Artful Meditation program combines skill development in mindfulness meditation and art appreciation in the beautiful and intimate context of an art gallery. Mindfulness meditation has two interrelated components: self-regulation of attention and the cultivation of attention that is non-judgmental, curious, and focused on the present moment.

Participants are first offered meditation instruction and taken through a guided meditation. Participants are then guided through a series of meditative art appreciation exercises detailed in Section 4 of this implementation guide in which the object of meditation shifts from their breath to an object of art in the gallery. The program closes with discussion and another brief period of mindful meditation.

There are five main components of the Artful Meditation program: (1) welcome and introduction; (2) mindful meditation; (3) meditative art appreciation; (4) discussion; (5) conclusion and mindful meditation. The program is about 1 hour and 15 minutes in duration.

**What?**

Materials

Artful Meditation should take place in a gallery, either in-person or online.

If in-person it should be closed to other visitors when possible to reduce distractions. The program involves two interconnected components that require two separate seating set ups:

**(1) Mindful meditation**: a large circle of seating should be created that consists of several different seating options to accommodate participants’ differing comfort levels including chairs, *zabutons* (or large square cushions that protect one’s buttock, knees, and ankles when sitting on the floor), and *zafus* (smaller circular cushions that are placed on top of the *zabutons*, and that can be stacked as needed to create enough height to sit comfortably on the floor).

**(2) Meditative art appreciation**: A museum stool for each participant (e.g., 20 stools required for 20 participants) should be placed in front of a work of art at a distance appropriate for the size of the work (i.e., closer for smaller objects, farther for larger objects/paintings). If there are not enough objects of art for each participant to have their own, then multiple stools can be placed around a single object of art.

In addition to these seating arrangements it can be helpful to have a meditation bell to start and conclude each activity.

If a virtual, or online, version of the Artful Meditation program is chosen, the facilitator should advise participants ahead of time to find a comfortable way to engage with the program on-screen, be that a chair, meditation cushion, or some other preferred seating. An upright, but comfortable, posture is desired.

In a virtual environment, the facilitator(s) will select an image of a piece of art and display it at the appropriate points in the program (detailed below). Rehearsals to ensure the integrity of the technology and facilitator comfort with the chosen platform are highly recommended for virtual programs.

Procedures

The five main components of the Artful Meditation program are detailed below.

1. **Welcome and introduction:**
2. Initial welcome (in the lobby or onscreen in a virtual program):
   - 1. For a live event, a welcome/check-in table can be placed near the front desk, and a sign placed near the main entrance informing Artful Meditation participants that they may bypass the front desk and proceed directly to the check-in table.
     2. Participants are greeted and given an Artful Meditation sticker, which gives them full access to the museum for the day. Participants gather as a group before being led together to the chosen gallery.
     3. Participants are asked to turn off or silence cell phones.
3. Once in the gallery space, seated together, (or after introductory guidance in a virtual program) the facilitator invites everyone to first take a few breaths together.
4. Participants are told that the program has been designed to be as comfortable as possible but that if they need anything (e.g., a restroom) they should feel free to let the facilitator(s) know.
5. Encourage participants to experiment with different seating options in order to find an upright, yet comfortable position.
6. Offer a brief overview and introduction to the program
   1. The goal of the program is to enjoy art, the museum space, and each other’s company while developing skills in mindful meditation and art appreciation
   2. No art experience is needed, there are no right or wrong answers, and everyone’s ideas and viewpoints are equally valid
   3. Review the program’s schedule/components and a brief explanation of the “slow looking” portion of the program - shifting one’s attention from focusing on the breath to focusing on a work of art.
7. Offer reminders about how to keep the art safe during the program (e.g., no pens, no water, be aware of your surroundings).
8. Inform participants that the program lasts about 1 hour and 15 minutes and that they are free to enjoy the museum on their own for the rest of the day as part of the program.
9. The Art Appreciation facilitator then offers a brief overview of the art on view in the gallery space. This overview should include some history of the artist or artists on view, some highlights of the art, information on what makes this art special, and some keys on what they might look for when they regard the art.
10. If a separate Meditation Facilitator is used, they can introduce themself and invite participants to do the same:
    - 1. Each participant is invited to say their name and preferred pronouns, their experience with meditation, and what they hope to get out of the program.
      2. Participants should be told before starting how much time they will have to respond to these prompts. We recommend no more than 15-30 seconds. We have found that this is enough time for everyone to have a chance to express themselves but also assures that other aspects of the program are not cut short.
11. **Mindful meditation instruction:**
    1. The facilitator provides an overview of mindful meditation and beginning meditation instruction:
       1. Mindful meditation involves two interconnected skills – self-regulation of attention and the cultivation of attention that is non-judgmental, curious, and focused on the present moment.
       2. Participants are again invited to find a *comfortable* and *upright* sitting posture. The sitting posture should ideally allow the participants’ hips to be higher, even if slightly, than their knees which will facilitate an upright spine that preserves its natural shape. In addition, individuals should sit such that they have a ‘tripod’ of support – either their buttock and knees if sitting on a cushion on the floor or their buttock and their feet if they are sitting on a chair.
       3. Participants are invited to either close their eyes or adopt a ‘gentle gaze’ at nothing in particular at about a 45-degree angle downwards. Keeping eyes open can be helpful if a person feels tired and closing one’s eyes can be helpful if one is feeling distracted or unsettled.
       4. Once participants have found a comfortable seated position they are offered basic meditation instruction: *rest one’s attention on the sensations of breathing;* allow one’s attention to follow the rhythmic sensations of each inhalation and exhalation. The *breath, therefore, becomes the object of meditation*.
       5. It is important to normalize the fact that it is natural for the mind to wander from this object of meditation to other aspects of one’s lived experience – physical sensations, emotions, sounds, etc. This is the reality of the human condition – we think, we feel, we touch, we hear, we taste, we smell, we see…in short, we are alive – it is not an indicator of being a ‘bad’ meditator or of being ‘unable to do it.’ Meditation is not about slipping away into a void. On the contrary, slowing down and paying attention to one’s life makes it more vivid. Emphasize that thinking and feeling are natural parts of the meditation experience.
       6. Instruct participants to simply note when they have left the breath and then gently and precisely return to it over and over again as needed. It can also be helpful to remind participants that self-regulation or concentration is only one part of meditation; the other part is cultivating a non-judgmental and curious quality of mind. ‘Distractions,’ whether they be pleasant, unpleasant, or neutral are a perfect opportunity to practice this critical element to meditation.
       7. Once you’ve given the basic meditation instruction ask if anyone has questions.
    2. Guided mindful meditation experience: The facilitator leads the group in a 10- to 15-minute guided meditation.
       1. Strike the bell three times to start the meditation and two times to conclude it offering encouraging prompts as needed. Prompts may include reminders to return to the breath, that nothing needs to be done, that all that is required in meditation is to simply rest one’s attention on the natural rhythm of the breath.
12. **Meditative art appreciation**
13. At the conclusion of the 2^nd^ bell the facilitator invites participants to carefully come to a standing position and slowly walk to one of the available stools positioned in front of an object of art. (In virtual programs, the facilitator invites participants to slowly come out of the meditation to rest their attention on the art object that is now being presented on their screens.
14. As participants are walking to the stools, in a live program, the facilitator suggests they not focus on what artwork to sit in front of, but instead to simply select a stool; the facilitator also suggests participants may want to keep their gaze lowered once they have taken their seat.
15. Once everyone is seated in front of an artwork, the facilitator invites them to look up at the artwork and to shift the focus of their attention from their breath to the object in front of them.
16. The facilitator then guides the group in a “slow-looking” exercise with the artwork for the next 12-15 minutes. Instructions are given slowly, with time in between for looking in silence. A quote either from an art critic or the artist is usually offered as a way to provide context and additional information about the intent behind the artwork. The goal is to help people give themselves permission to just focus on this one work of art and to notice all the details that the artist put into the work.
17. Invite participants to return to the circle (or, in a virtual program) remove the view of the object, return the facilitator to the screen, and request that everyone re-focus for discussion).
18. **Discussion**
19. Once seated again in the circle, participants are invited to form groups of 2 or 3 and share with their group one thing that surprised them during the guided looking exercise. Give participants approximately 5 minutes for this discussion. (Note: this step can be omitted in virtual programs.)
20. The facilitator(s) then lead a whole-group discussion by saying, “We now want to take some time for discussion. This is a chance to share anything you would like about your experience in either the meditation or art appreciation components of today’s program.”
21. **Conclusion and mindful meditation**
22. Thank everyone for coming.
23. Encourage everyone to try the “slow looking” exercise on their own whenever they like at the museum or in daily life.
24. Remind participants of the date of the next program (if relevant) and encourage them to attend.
25. If time remains the facilitator can lead the group in another period of mindful meditation practice, again starting it with three strikes of the bell and concluding it with two.

**Who Provided?**

This program can be facilitated by either a solo leader or by two co-facilitators, depending on the skills and experiences of the leader(s). Whether facilitated solo or in tandem, the following are suggestions for knowledge and skills required to effectively lead an Artful Meditation program.

**Art Appreciation:** The facilitator should be familiar with the museum’s art collection, have formal training in art or museum studies, and be at least somewhat familiar with mindful meditation.

**Meditation:** It is recommended that the facilitator has over 5 years of experience teaching mindful meditation practices to diverse groups in varied settings and have at least 10 years of an ongoing personal meditation practice.

Facilitators leading virtual programs must be practiced in the use of the selected online forum and be able to, for example, assist participants with getting online and adjusting their audio and video settings. Prior to leading their first Artful Meditation program it is suggested that facilitators, or co-facilitators, observe a minimum of one (two is preferred) Artful Meditation programs, either live or online, and that they lead one (two is preferred) mock Artful Meditation programs to be evaluated by the program administrator and a minimum of one other museum staff.

1. Mock tours will be composed of approximately five to ten individuals standing in as Artful Meditation guests.
2. Meet with evaluators after the mock program for feedback and acceptance, rejection or contingent approval as an Artful Meditation facilitator.
3. Those who receive contingent approval may be required to lead another mock Artful Meditation program or be able to address specific criticisms before acceptance into the program as an Artful Meditation facilitator.

**How?**

Artful Meditation programs are either in-person group experiences in a museum’s gallery space, or virtual online programs accessed via a museum or institutional web platform. Group size varies. We recommend no less than 4 participants and no more than 20 participants in a group.

**Where?**

Artful Meditation programs can take place at any type of art museum, gallery, or institution, or programs can be virtual, hosted on a platform such as Zoom, Google Groups, or another online forum. Facilitators leading virtual programs must be practiced in the use of the selected online forum and aware of the importance of good lighting and audio fidelity. Rehearsals to ensure the integrity of the technology and all audio/visual issues are highly recommended for virtual programs.

**When and How Much?**

Artful Meditation programs can be scheduled as frequently or as rarely as staff and resources permit. Consider the needs of your participants. Scheduling programs on both weekdays and weekends can accommodate the varying routines and obligations of participants. Unless the demand for Artful Meditation programs becomes extremely high, there is typically no need to limit how many programs an individual can attend.

**Modifications**

Each Artful Meditation program takes place in a different gallery with different art works selected by the facilitator. Selecting different objects of art for each program helps assure the program’s diversity and inclusivity by appealing to the widest possible range of art. Artful Meditation programs should be publicized well in advance (i.e., at least a month) prior to any particular program.

**How Well?**

It is recommended, but not required, that every Artful Meditation program be evaluated in a structured way, using a “Fidelity Checklist.” This can provide invaluable data on which to base refinements to the program, its content, and its presentation. An assistant to the facilitator, or facilitators, could use the Artful Meditation Fidelity Checklist to record adherence to recommended steps and processes.

**ARTFUL MEDITATION – FIDELITY CHECKLIST**

**Completed by:**

**Date:**

1. **Welcome**

- Research partnership with UC Davis introduced
- Program goal reviewed
- Program schedule/components reviewed
- There are no right or wrong answers
- Participants are encouraged to find a comfortable sitting position. We want you to be as comfortable as possible so please feel free to get up and move if you need to during the program.
- Participants are encouraged to ask for help if needed
- Participants informed that the program lasts about 1 hour and 15 minutes

**Program Introduction**

- The Art Appreciation facilitator offered an overview of the program and a short 5-10 minute presentation on the art/artists that were the focus of the day’s program.
- The Meditation Facilitator created an opportunity for everyone to introduce themselves:
  - 15-30 seconds per participant: (1) their name, (2) their experience with meditation and (3) what they hope to get out of the program

*The Artful Meditation facilitator(s) introduced participants to the program’s goal?*

YES / NO

*The Artful Meditation facilitator(s) was welcoming?*

YES / NO

1. **Mindful meditation instruction:**

- Overview of mindful meditation: self-regulation of attention and the cultivation of attention that is non-judgmental, curious and focused on the present-moment.
- Body position: comfortable seated position; eyes closed or at 45-degree angle with gentle gaze
- Meditation instruction: rest one’s attention on the sensations of breathing; the breath becomes the object of meditation
  - natural for the mind to wander and to continue to experience physical sensations, emotions, sounds, etc.
  - this awareness of the lived experience is not an indicator of being a ‘bad’ meditator or of being ‘unable to do it.’
  - emphasize that thinking and feeling are a natural part of the meditation experience
- Instruct the participants to simply note when they have left the breath and then to gently and precisely return to it over and over again as needed.
- See if anyone has questions
- Guided mindful meditation experience: The meditation facilitator led the group in a 10-15-minute guided meditation.
- Encouraging prompts offered as needed – e.g. return to the breath, nothing needs to be done, all that is required in meditation is to simply rest one’s attention on the natural rhythm of the breath.

*The Meditation Facilitator introduced participants to mindful meditation in a friendly and encouraging manner that included responding to participants’ questions.*

YES / NO

1. **Meditative art appreciation**

- The Art Appreciation facilitator invited participants to shift the focus of their attention from their breath to the artwork in front of them
- The facilitator led participants in a 12-15 minute guided slow-looking exercise
- The slow-looking exercise noted particular aspects of the artworks
- A quote from a critic or artist is offered to add information and context
- Time is offered between instructions for silent exploration of the artwork

*The Art Appreciation Facilitator offered a guided slow-looking exercise in a friendly and encouraging way*

YES / NO

1. **Discussion**

- The Art Appreciation and Meditation Facilitators led the group in a discussion
- Participants were encouraged to say anything they would like to about their experience during either the meditation and/or art appreciation components of the program
- Facilitators acknowledged and responded to each participant comment

*The facilitators led a group discussion, encouraging participation and validating all comments*

YES / NO

1. **Conclusion and mindful meditation**

- Facilitator(s) thanked everyone for coming
- Facilitator(s) reminded participants of the date of the next program and encouraged them to attend
- Meditation Facilitator led the group in another period of mindful meditation, time permitting

The facilitator(s) concluded the program

YES / NO

**References**

1. Clift S, Camic PM. *Oxford Textbook of Creative Arts, Health, and Wellbeing.* Oxford University Press; 2016.

2. Fancourt D, Finn S. What is the evidence on the role of the arts in improving health and well-being? A scoping review. *Copenhagen: WHO Regional Office for Europe.* 2019.

3. Rice AS, Smith BH, Blyth FM. Pain and the global burden of disease. *Pain.* 2016;157(4):791-796.

4. Institute of Medicine. *Relieving pain in America: A blueprint for transforming prevention, care, education, and research.* National Academies Press;2011.

5. Merskey H, Bogduk N. Task force on taxonomy of the International Association for the Study of Pain: Classification of chronic pain. Description of pain syndromes and definitions of pain terms. In: IASP Press, Seattle, WA; 1994.

6. Turk DC, Monarch ES. Biopsychosocial perspective on chronic pain. *Psychological approaches to pain management: A practitioner’s handbook.* 1996:3-32.

7. Gatchel RJ, Peng YB, Peters ML, Fuchs PN, Turk DC. The biopsychosocial approach to chronic pain: scientific advances and future directions. *Psychological bulletin.* 2007;133(4):581.

8. Blyth FM, Macfarlane GJ, Nicholas MK. The contribution of psychosocial factors to the development of chronic pain: The key to better outcomes for patients? *Pain.* 2007;129(1):8-11.

9. Hadjistavropoulos T, Craig KD, Duck S, et al. A biopsychosocial formulation of pain communication. *Psychological bulletin.* 2011;137(6):910.

10. Raja SN, Carr DB, Cohen M, et al. The revised International Association for the Study of Pain definition of pain: concepts, challenges, and compromises. *Pain.* 2020;161(9):1976-1982.

11. Master SL, Eisenberger NI, Taylor SE, Naliboff BD, Shirinyan D, Lieberman MD. A Picture's Worth Partner Photographs Reduce Experimentally Induced Pain. *Psychol Sci.* 2009;20(11):1316-1318.

12. Younger J, Aron A, Parke S, Chatterjee N, Mackey S. Viewing pictures of a romantic partner reduces experimental pain: Involvement of neural reward systems. *PLoS One.* 2010;5(10):e13309.

13. Eisenberger NI, Master SL, Inagaki TK, et al. Attachment figures activate a safety signal-related neural region and reduce pain experience. *Proceedings of the National Academy of Sciences.* 2011;108(28):11721-11726.

14. Hadjistavropoulos T, Craig KD, Duck S, et al. A biopsychosocial formulation of pain communication. *Psychol Bull.* 2011;137(6):910-939.

15. Camic PM, Chatterjee HJ. Museums and art galleries as partners for public health interventions. *Perspectives in public health.* 2013;133(1):66-71.

16. Fancourt D, Steptoe A. Physical and Psychosocial Factors in the Prevention of Chronic Pain in Older Age. *The Journal of Pain.* 2018.

17. Slayton SC, D’Archer J, Kaplan F. Outcome studies on the efficacy of art therapy: A review of findings. *Art Ther.* 2010;27(3):108-118.

18. Craig P, Dieppe P, Macintyre S, Michie S, Nazareth I, Petticrew M. Developing and evaluating complex interventions: the new Medical Research Council guidance. *BMJ.* 2008;337:a1655.

19. Hoffmann TC, Glasziou PP, Boutron I, et al. Better reporting of interventions: template for intervention description and replication (TIDieR) checklist and guide. *BMJ.* 2014;348:g1687.
